# Transdiagnostic culturally adapted CBT with Farsi-speaking refugees: a pilot study

**DOI:** 10.1080/20008198.2017.1390362

**Published:** 2017-11-07

**Authors:** Schahryar Kananian, Sarah Ayoughi, Arieja Farugie, Devon Hinton, Ulrich Stangier

**Affiliations:** ^a^ Department of Psychology, Goethe-Universität Frankfurt, Frankfurt am Main, Germany; ^b^ Department of Psychiatry, Harvard Medical School, Boston, USA

**Keywords:** Traumatized refugees, trauma, culturally adapted cognitive behavioural therapy, Afghanistan, transdiagnostic treatment, group treatment, Refugiados traumatizados, trauma, Terapia cognitivo-conductual adaptada culturalmente, Afganistán, tratamiento transdiagnóstico, tratamiento en grupo, 受创伤的难民, 创伤, 文化适应认知行为疗法, 阿富汗, 跨诊断治疗, 群体治疗, • Feasibility of a transdiagnostic CBT demonstrated for Farsi-speaking refugees. • Cultural adaptation of CBT for Farsi-speaking refugees in respect to symptom perception. • causal attributions, treatment expectations, and ideas of healing. • Group setting increases social support and reduces economic cost.

## Abstract

**Background**: Approximately half of all asylum seekers suffer from trauma-related disorders requiring treatment, among them Posttraumatic Stress Disorder (PTSD), depression, anxiety, and somatic symptoms. There is a lack of easily accessible, low-threshold treatments taking the cultural background into account. Culturally Adapted CBT (CA CBT) is a well evaluated, transdiagnostic group intervention for refugees, using psychoeducation, meditation, and Yoga-like exercises. **Objective:** An uncontrolled pilot study with male Farsi-speaking refugees from Afghanistan and Iran was conducted to investigate feasibility with this ethnic group; a group for which no previous CBT trials have been reported.

**Method**: The participants were nine Farsi-speaking, male refugees with M.I.N.I./DSM-IV diagnoses comprising PTSD, major depressive disorder, and anxiety disorders. Treatment components were adapted to the specific cultural framework of perception of symptoms, causes, ideas of healing, and local therapeutic processes. Before and after 12 weeks of treatment, the primary outcome was assessed using the General Health Questionnaire (GHQ-28). Secondary outcome measures were the Posttraumatic Checklist, Patient Health Questionnaire, Somatic Symptom Scale, World Health Organization Quality of Life Questionnaire (WHOQOL-BREF), Affective Style Questionnaire (ASQ), and Emotion Regulation Scale (ERS).

**Results**: Seven participants completed treatment. In the completer analysis, improvements were found on almost all questionnaires. Large effect sizes were seen for the GHQ-28 (*d* = 2.0), WHOQOL-BREF scales (*d* = 1.0–2.3), ASQ tolerating subscale (*d* = 2.2), and ERS (*d* = 1.7). With respect to feasibility, cultural adaptation seemed to be a crucial means to promote effectiveness.

**Conclusion**: CA CBT may reduce general psychopathological distress and improve quality of life. Improvement in emotion regulation strategies may mediate treatment effects. More support should be provided to enhance coping with the uncertainty of asylum status and stressful housing conditions. CA CBT appears to be a promising transdiagnostic treatment, serving as an initial low-threshold therapy in a stepped care approach.

## Abstract (es)

**Planteamiento**: Aproximadamente la mitad de los solicitantes de asilo sufren trastornos relacionados con el trauma que requieren tratamiento, entre ellos trastorno por estrés postraumático (TEPT), depresión, ansiedad y síntomas somáticos. Hay una falta de tratamientos de fácil acceso y umbral bajo que tienen en cuenta los antecedentes culturales. La TCC adaptada culturalmente (TCC AC) es una intervención grupal transdiagnóstica bien evaluada para los refugiados, que utiliza ejercicios de psicoeducación, meditación y de Yoga.

**Objetivo:** Se realizó un estudio piloto no controlado con hombres refugiados de habla farsi de Afganistán e Irán para investigar la viabilidad con este grupo étnico, grupo para el cual no existen informes de ensayos previos de TCC.

**Método**: Los participantes fueron nueve refugiados varones de habla farsi, con diagnósticos M.I.N.I./DSM -IV que comprenden TEPT, trastorno depresivo mayor y trastornos de ansiedad. Los componentes del tratamiento se adaptaron al marco cultural específico de percepción de síntomas, causas, ideas de curación y procesos terapéuticos locales. Antes y después de 12 semanas de tratamiento, el resultado principal se evaluó mediante el Cuestionario de Salud General (GHQ-28, siglas en inglés de General Health Questionnaire). Las medidas de resultado secundarias fueron la Lista de Verificación Postraumática (Posttraumatic Checklist), el Cuestionario de Salud del Paciente (Patient Health Questionnaire), la Escala de Síntomas Somáticos (Somatic Symptom Scale), el Cuestionario de Calidad de Vida de la Organización Mundial de la Salud (WHOQOL-BREF, siglas en inglés de World Health Organization Quality of Life Questionnaire), el Cuestionario de Estilo Afectivo (ASQ, siglas en inglés de Affective Style Questionnaire), y la Escala de Regulación Emocional (ERS, siglas en inglés de Emotion Regulation Scale).

**Resultados**: Siete participantes completaron el tratamiento. En el análisis más completo, se encontraron mejoras en casi todos los cuestionarios. Se observaron tamaños de efecto grandes para las escalas GHQ-28 (*d* = 2,0), WHOQOL-BREF (*d* = 1,0–2,3), la subescala de tolerancia ASQ (*d* = 2,2) y ERS (*d* = 1,7). Con respecto a la viabilidad, la adaptación cultural parece ser un medio crucial para promover la eficacia.

**Conclusión**: La TCC AC puede reducir la angustia psicopatológica general y mejorar la calidad de vida. La mejora en las estrategias de regulación emocional puede mediar en los efectos del tratamiento. Debería prestarse más apoyo para mejorar la capacidad de afrontar la incertidumbre sobre el estado de asilo y condiciones de vivienda estresantes. La TCC AC parece ser un tratamiento transdiagnóstico prometedor, que sirve como una terapia de bajo umbral inicial en un abordaje de atención escalonada.

## Abstract (zh)

**背景：**大约有一半的避难者承受着需要治疗的创伤相关的疾病，这些疾病包括创伤后应激障碍（PTSD），抑郁，焦虑和躯体化症状。很少有易得的、低门槛的治疗方法考虑到文化差异的问题。文化适应CBT（CA CBT）是一种经过良好评估、跨诊断的难民群体干预方法。这一方法包括了心理教育、冥想和类瑜伽锻炼的内容。

**目标：**一个未经控制的初步研究针对来自阿富汗和伊朗的说波斯语的男性难民，以考察在这个种族群体中的可行性。而之前还没有任务这个群体中 关于CBT试验的报告。

**方法：**参与者是9名说波斯语的男性难民，经过M.I.N.I./DSM-IV诊断表现出了PTSD，重度抑郁症和焦虑症。专门针对文化框架对治疗方案进行了调整，包括对症状的认识，原因，治愈的想法和本地化咨询过程。在治疗之前和治疗后12周使用一般健康问卷（GHQ-28）对主要结果进行了测查。次级结果测量使用创伤后检查表，患者健康问卷，躯体化症状量表，世界卫生组织生活质量问卷（WHOQOL-BREF），情感风格问卷(ASQ)和情绪控制量表(ERS)。

**结果：**7名参与者完成了治疗，对完成者的分析发现，在所有问卷上均能够看到提高。在以下问卷上能够看到很大的效果量：在GHQ −28(*d* = 2.0)，在WHOQOL-BREF问卷(*d* = 1.0-2.3)，ASQ忍耐分量表上(*d* = 2.2)，ERS上(*d* = 1.7)。就可行性而言，文化适应看起来是提升效果的重要方法。

**结论：**CA CBT可能减少一般精神病理学的痛苦，并提升生活质量。情绪控制策略的提升是治疗效果的中介因素。应该提供更多地支持，帮助他们提高对不确定的避难状态和有压力的居住环境的应对能力。CA CBT看起来是一种有希望的跨诊断性治疗方案，可以作为一种逐级关怀方案中初始的低门槛治疗方法。

## 1. Introduction

Most of the refugees who came to Germany within the last three years have experienced traumatic events including combat, terror attacks, imprisonment, torture, kidnapping, or rape (Richter, Lehfeld, & Niklewski, 2015). In addition, flight itself is associated with life-threatening and other traumatic experiences. Furthermore, placement in provisional collective housing and insecure outcome of the asylum proceedings further contribute to the accumulation of distress and to the maintenance and aggravation of mental disorders (Fazel & Wheeler, 2005; Sulaiman-Hill & Thompson, 2012). Epidemiological studies indicate that about half of the refugees in reception centres suffer from mental disorders that need to be treated (Heeren et al., 2014; Richter et al., 2015), among them posttraumatic stress disorder (PTSD; 21–54%), depression (20–56%), anxiety disorders (40–56%), and somatic symptoms without medical explanation (37%; Rohlof, Knipscheer, & Kleber, 2014).

**Figure 1.**
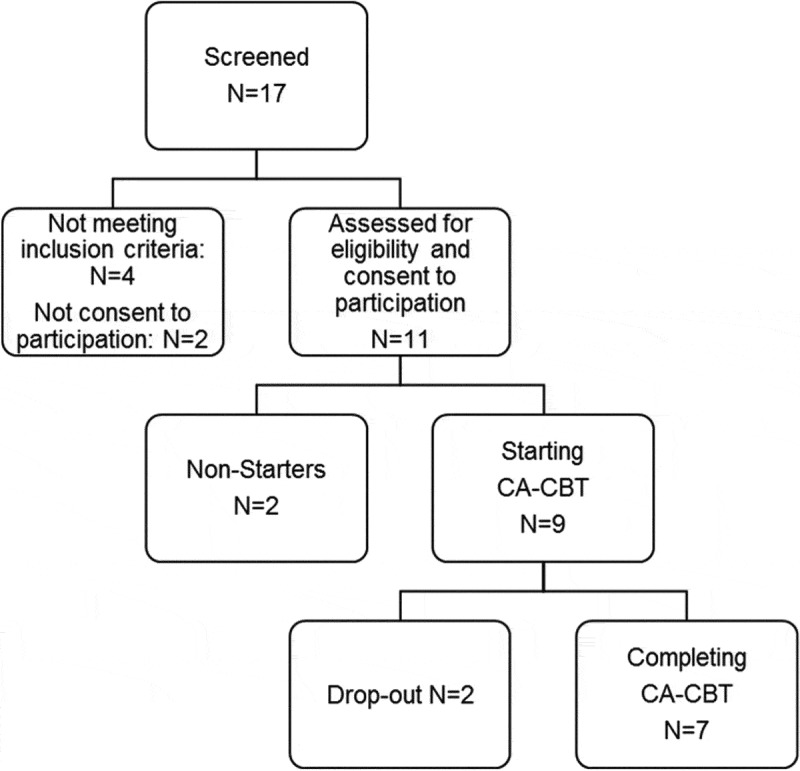
Flowchart of participants.

About 12% of the refugees who have applied for asylum in Germany in 2016 come from Afghanistan (Bundesamt für Migration, 2016). The large majority of the Afghan population endured multiple traumatic events over many years. A population-based mental health survey (Lopes Cardozo et al., 2004) revealed elevated rates of PTSD (42.1%), symptoms of depression (67.7%), and symptoms of anxiety (72.2%). Using the General Health Questionnaire (GHQ-28), several studies demonstrated high prevalence rates of psychiatric conditions in Afghan refugees (Azizi, Holakoie Naieni, Rahimi, Amiri, & Khosravizadegan, 2005; Kalafi et al., 2002; Sadeghi, Shojaeizadeh, Arefi, & Shaahmadi, 2016). Despite the high prevalence of mental health problems, Western concepts of psychopathology such as the concept of PTSD or depression are hardly known in Afghanistan (Alemi, Weller, Montgomery, & James, 2017; Yaser, Slewa-Younan, Smith, Olson, & Guajardo, 2016). One study found that Afghan refugees considered ‘improving diet’ as more helpful than seeing a psychologist in treating PTSD (Yaser et al., 2016). CBT principles may be less easily understood, suggesting the need for techniques like somatic-based emotion regulation that are easily explained and learned (Hays, 2009; Hinton & Patel, in press; Hinton, Rivera, Hofmann, Barlow, & Otto, 2012; Jackson, Schmutzer, Wenzel, & Tyler, 2006). Complaints may differ from Western groups such as local idioms of distress and a much greater emphasis on somatic distress (Alemi et al., 2017; Hinton & Patel, in press; Hinton et al., 2012). Thus, there are important reasons to culturally adapt CBT in a way that psychotherapy is easier accessible for people from non-Western cultures.

Culturally Adapted CBT (CA CBT) is a transdiagnostic treatment approach developed by Hinton et al. (2011), adapted for the treatment of PTSD among traumatized refugees and ethnic minority populations. It includes psychoeducation on PTSD, depression, anxiety disorders, and anger; a focus on somatic sensation including exposure and re-association; somatic-focused techniques like stretching and Yoga exercises; and multiple emotion regulation techniques such as meditation and somatic-focused techniques like those mentioned above. Of note, CA CBT does not include prolonged exposure to trauma memories but rather addresses trauma symptoms and ways of dealing with them. Changes in emotion regulation have been identified as potential mediators of the effects of CA CBT (Hinton et al., 2012) and include increasing acceptance of and distancing from negative thoughts and affects and enhancing reappraisal of negative experiences. Emotion regulation is taught in CA CBT by such means as imagery techniques, mindfulness, loving kindness meditation, and applied stretching. CA CBT has proven its effectiveness with various cultural groups, for example, Cambodians, Egyptians, Latinos, South African tribal groups, and Vietnamese (Hinton et al., 2005; Hinton, Hofmann, Pollack, & Otto, 2009, 2011, in press; Jalal, Samir, & Hinton, 2017).

To our knowledge, no studies have been conducted investigating psychological treatments for traumatized Farsi-speaking refugees such as Afghans. As indicated above, CA CBT is culturally adapted for such groups in various ways: by providing psychoeducation, addressing somatic distress (e.g. modifying catastrophic cognitions about those complaints), and teaching easily understood techniques such as somatic-based emotion regulation and mindfulness. We further adapted the treatment to this ethnic group by including examples and metaphors from the Afghan culture. We used a group setting to facilitate social support, reduce stigma, and reduce catastrophic cognitions about symptoms (Sulaiman-Hill & Thompson, 2012); also, a group treatment increases scalability and hence public health impact. We expected the treatment to reduce general psychopathology as assessed by the GHQ-28; to improve quality of life as assessed by WHOQOL scales; and to improve emotion regulation ability as assessed by ACQ and the ERS, with emotion regulation hypothesized to be a key mediator of treatment effect.

## 2. Method

### 2.1. Participants

The participants were recruited from the metropolitan area of Frankfurt via flyers and posters in refugee camps or were referred to the Counseling Center for Refugees at the Goethe University. For the flowchart of participants see Figure 1. Inclusion criteria were: (1) a DSM-5 diagnosis of Trauma- and Stressor-Related Disorders, Depressive Disorders, Anxiety Disorders, or Somatoform Symptoms and Related Disorders as confirmed by the *Mini-International Neuropsychiatric Interview 7.0* for DSM-5 *(M.I.N.I.*: Hergueta & Weiller, 2013; Sheehan, Lecrubier, & Sheehan, 1998), (2) a GHQ-28 score of at least 11, (3) native language being Farsi (or Dari), (4) being older than 18 years, and (5) being male. The reasons for the inclusion of only male participants were potential cultural barriers to discussing sensitive topics between men and women. Exclusion criteria included imminent suicide risk, acute psychotic episode, personality disorders from the dramatic cluster, or addiction. A total of nine male participants were enrolled in the group treatment. Seven were from Afghanistan, two from Iran. The mean age was 25.6 (*SD* = 9.0). All except one participant were single, and all had completed elementary school. Only one participant had employment, a part-time job. Three participants suffered from sustained injuries as long-term consequence of torture. The diagnoses measured by the M.I.N.I. were as follows: PTSD (*N* = 7), Major Depression (*N* = 5), Panic Disorder (*N* = 2), Generalized Anxiety Disorder (*N* = 2).

### 2.2. Procedure

The study was carried out between October 2016 and February 2017 in the Behavior Clinic of the Department of Psychology at Goethe University Frankfurt. Diagnostic interviews and pre- and post-assessments were conducted by an independent, Farsi-speaking postgraduate psychologist who assisted self-ratings when needed. Treatment was delivered in a group and conducted by two Farsi-speaking therapists. One had a master’s degree, and the other PhD in Clinical Psychology with extensive experience in conducting field studies within Afghanistan and training psychosocial counsellors within the Afghan Health System. The weekly supervisions were done with a trained supervisor in Clinical Psychology and the professor for Clinical Psychology at the Goethe-Universität Frankfurt.

### 2.3. Measures

Farsi versions of all instruments were used. The GHQ-28, the WHOQOL, and the ASQ have been validated in Farsi (see below). For the other questionnaires, the original versions were translated and back-translated by different native Farsi speakers and discrepancies clarified, in accordance with standard procedure (van Ommeren et al., 1999).

#### 2.3.1. General Health Questionnaire (GHQ-28)

The GHQ-28 (Goldberg et al., 1997) was developed as a screening tool to detect those who are likely to have or to develop a psychiatric disorder. It consists of 28 items measured on a 4-point Likert scale ranging from 0 to 3. The GHQ-28 is divided into four subscales: (1) somatic symptoms, (2) anxiety/insomnia, (3) social dysfunction, and (4) severe depression. The instrument has been validated, showing excellent psychometric properties, and has been extensively used in studies investigating mental health in Afghan refugees (Azizi et al., 2005; Kalafi et al., 2001; Kalafi, Hagh-Shenas, & Ostovar, 2002; Sadeghi et al., 2016). The GHQ-28 was defined as the primary outcome.

#### 2.3.2. Posttraumatic Checklist for DSM-5 (PCL-5)

The PCL-5 measures the five clusters of the DSM-5 for PTSD (Weathers et al., 2013). It consists of 20 items measured on a 5-point Likert scale ranging from 0 to 4. It has high test-retest reliability and high convergent and discriminant validity (Blevins, Weathers, Davis, Witte, & Domino, 2015).

#### 2.3.3. Patient Health Questionnaire (PHQ-9)

The PHQ-9 (Kroenke, Spitzer, & Williams, 2001) is a reliable and valid measure of depression severity whose nine items refer to the DSM-IV criteria for Major Depression. Items are measured on a 4-point Likert scale ranging from 0 to 3.

#### 2.3.4. Somatic Symptom Scale (SSS-8)

The SSS-8 is used to assess somatic symptoms. It consists of eight items and it has been largely validated (Gierk et al., 2014). It has a high content validity and high construct validity and high Cronbach’s α (Gierk et al., 2014; Zijema et al., 2013).

#### 2.3.5. World Health Organization Quality of Life Scale Questionnaire (WHOQOL-BREF)

The WHOQOL-BREF (Skevington, Lotfy, & O’Connel, 2004) is a cross-culturally comparable instrument to assess the quality of life. It consists of 26 items measured on a 5-point Likert scale. It is divided into four domains: (1) physical health, (2) psychological health, (3) social relationships, and (4) environment. A higher score indicates better quality of life. All domains have an acceptable Cronbach’s α and high discriminant validity in Farsi (Nedjat, Montazeri, Holakouie, Mohammad, & Majdzadeh, 2008).

#### 2.3.6. Affective Style Questionnaire (ASQ)

The ASQ (Hofmann & Kashdan, 2010) contains 20 items on a 5-point Likert scale, measuring three emotion regulation styles: (1) concealing, (2) adjusting, and (3) tolerating. Higher scores reflect a preference for an affective style. The internal consistency values are satisfying, convergent and discriminant validity was demonstrated by associations between existing instruments that measure similar constructs. The ASQ has also been validated in Farsi language (Badri, Vahedi, Bairami, & Einipour, 2015).

#### 2.3.7. Emotion Regulation Scale (ERS)

The ERS (Hinton et al., 2009) is a 10-item scale, each item rated on a 0–4 Likert scale assessing the ability to distance from dysphoric affects, higher scores indicating greater ability to distance from negative affect. The measure has shown good test-retest reliability and high internal consistency in samples of Cambodian refugees with PTSD (Hinton et al., 2009) and female Latino patients with treatment-resistant PTSD (Hinton et al., 2011).

### 2.4. Intervention and cultural adaptation

The group programme was based on the Manual for Culturally Adapted Cognitive Behavioral Therapy (Hinton et al., 2012). The original manual includes 14 sessions, but for the current study the number was reduced to 12 sessions. Refugees in Germany have unsettled life situations in terms of work and residence, so the number of sessions was reduced to avoid non-attendance. Based on the analyses of individual problems assessed before treatment, two sessions focusing on interoceptive exposure as well as two sessions focusing on somatic complaints were each merged into one.

The group generally met once a week for 12 successive weeks and each session lasted approximately 90 minutes. The structure and content was based on the manual used by Hinton and colleagues, who have applied these techniques for refugees from Cambodia and Vietnam for Latino groups, for Egyptians, and for South African tribal groups (Hinton et al., 2005, 2009; Jalal et al., in press, 2017). In each session, there was psychoeducation: symptoms related to depression, anxiety disorders, and PTSD were discussed with the participants. To address catastrophic cognitions, explanatory models or symptom descriptions used by the participants were used to enhance understanding; the treatment elicits patient understanding of symptoms, which allows this intervention. Prominent idioms of distress such as cultural syndromes and somatic symptoms gathered in advance were discussed in group (Alemi, James, & Montgomery, 2016; Miller et al., 2006; Yaser et al., 2016). Over the sessions participants were taught Yoga (stretching) exercises, and basic techniques of mindfulness. The session-by-session treatment outline with the primary topics covered during the classes is presented in Table 1.

**Table 1.** CA CBT sessions.

Session name and content
Session 1: psychoeducation about trauma-related disorder and introduction of emotional regulation techniques
Session 2: applied stretching and the toe-to-head muscle relaxation with visualization
Session 3: review of the toe-to-head muscle relaxation with visualization and the introduction of the anxiety protocol
Session 4: psychoeducation on trauma and introduction of the trauma recall protocol
Session 5: psychoeducation on trauma-related disorder, modification of catastrophic cognitions, and teaching emotional distancing
Session 6: interoceptive exposure, introduction to breathing exercises, head rotation, hyperventilation
Session 7: breathing exercises and relaxation techniques
Session 8: sleep disturbance, sleep paralysis, nocturnal panic
Session 9: worry and generalized anxiety disorder
Session 10: anger and anger protocol, psychoeducation on anger
Session 11: somatic complaints, assess and treat somatic symptoms
Session 12: cultural syndromes and final review

Further cultural adaptation of the treatment was based on results from empirical studies as well as personal experiences of the therapists in treatment and counselling with this population (Ayoughi, Missmahl, Weierstall, & Elbert, 2012). In addition, the individual patients’ perspective of mental health problems was assessed during one hour open interviews prior to beginning the study. Although answers were not analysed systematically by using qualitative methods, we discussed in the work group, in addition to the individual case conceptualization, general cultural aspects of Farsi-speaking refugees that should be incorporated into the manual.

Adaptations of the manual specific to the Afghan culture covered four dimensions:

1. Perception and appraisal of symptoms: Among Farsi speakers, idioms of distress include ‘asabi’ (nervous agitation), ‘gham’ (sadness), and ‘jigar khun’ (expression often used to describe grief after the loss of family members or friends) (Miller et al., 2006). Depression is often described as ‘thinking too much’ (ruminative sadness), ‘inability to perform daily tasks’, ‘abdominal pain’, ‘going crazy’, ‘goshagary’ (self-isolation), and ‘dementia’ (Alemi et al., 2017). Consequently, we modified or adapted expressions in sessions when disorder-specific symptoms were introduced. We also tried to reduce the fear of ‘going crazy’ by normalizing the meaning of symptoms.
2. Explanation of causes: Afghan refugees often report housing/accommodation related difficulties, loss of culture and identity, ‘thinking too much’, and financial hardship as explanations of depression (Alemi et al., 2016). Based on previous studies and on our own pre-treatment interviews, we incorporated these models on causes in disorder-specific psychoeducation.
3. Utilization of local therapeutic practices and concepts: Behaviours of Afghans that improve resilience include praying and seeking support in the family (Ayoughi et al., 2012). Common concepts to promote healthy behaviours also were ‘being together with family’, ‘socialization with people of similar age’, ‘acceptance of one’s life situation (as Will of God)’. In keeping with these ideas, we supported exchange with peers and the Iranian/Afghan community in general (session 12). Finally, mindfulness was implemented in a more spiritual way (sessions 5, 7, 9), since meditation as a Buddhist tradition is not accepted in Islamic culture.

## 3. Instructions to alter specific interventions in CA-CBT

- Religious references to the Quran were deleted because two participants were of Christian belief and three Muslim participants declared that they do not practice their faith. Also, some of the participants reported bad memories and distress in association with their religion. This was adapted in advance, after the religiosity was administered throughout the pre-assessment.
- The metaphor of the inner child was replaced by the metaphor of an alarm system. The reason for this was the comparison of the mental problems of adults with fears of a little child, which might cause irritation due to the specific role of males rooted in the Afghan culture. A further reason was that the inner child might not be understood as a part of the personality, which is a common metaphor in the Western world. This was modified after discussions within our group about the experiences of one coauthor (S.A.) who had worked with refugees, in Afghanistan and who had met similar difficulties when using this term.
- The contents of the visualization exercise were adapted to the Afghan culture. Whereas in CA CBT either of two visualizations are used (a lotus flower with Asian patients or a palm tree with Egypt participants), we found that a ‘bagh’ (garden) fit better for Afghan participants. In Iran/Afghanistan a garden or parks in general are a place or environment representing a quiet and familiar place. This was adapted during the group treatment when participants were asked which places they considered as calming and joyful; consequently the ‘bagh’ was used as the best place to visualize.
- Modification of loving kindness. In CA CBT, most sessions end with a meditation exercise. Several involve ‘loving kindness meditation’ (LKM). Patients had a hard time projecting loving kindness to all beings because ‘bad things the world had done’ to them. Therefore, LKM was changed to focus more on benevolence and compassion for oneself, family members, and friends.

## 4. Results

Two participants dropped out after the first session they attended: first session for one was session 2, the other session 5. The reasons for quitting were acute attacks of panic in one patient and the decision to change treatment setting in the other patient. Table 2 represents symptom score means at the two assessment periods for the completer sample (*N* = 7) for the GHQ-28, PHQ-9, PCL-5, SSS-8, ASQ, and ERS. Shapiro-Wilk-Test was used to test for normal distribution. Because tests for normal distribution do not perform well in small sample sizes, we computed Wilcoxon test for nonparametric data in paired samples. Within-group effect sizes at post-test were calculated using Cohen’s *d* (Cohen, 1988), with Cohen’s *d* = *M*_1_ – *M*_2_/s_pooled_. Table 2 presents the descriptive statistics and results in Wilcoxon signed-rank tests. In addition, effect sizes were calculated for completers (*N* = 7) and the intent-to-treat sample (ITT, *N* = 9).

**Table 2.** Pre- and post-treatment comparison (completer participants, *N* = 7).

	Pre-treatment M(SD)	Post-treatment M(SD)	Effect size Cohen’s *d* for Completer/ITT	Wilcoxon-Signed-Rank-Test
GHQ-28 total score	44.6(8.4)	25.8(8.7)	2.0/0.6	−2.4**
GHQ-28 subscale Somatic symptoms	9.6(3.4)	5.4(2.5)	1.4/0.7	−1.7
GHQ-28 subscale Anxiety/insomnia	14.4(2.6)	7.1(2.1)	3.0/1.5	−2.4**
GHQ-28 subscale Social dysfunction	8.9(3.9)	5.6(2.2)	1.0/0.1	−2.4**
GHQ-28 subscale Severe depression	9.4(3.8)	6.4(2.2)	1.0/0.7	−1.5**
PCL-5	48.8(14.1)	34.8(19.4)	0.8/0.2	−1.7
PHQ-9	15.7(6.4)	11.6(5.7)	0.6/0.5	−1.4
SSS-8	13.5(6.3)	13.2(5.8)	0.1/0.6	−0.3
WHOQOL Physical health	17.2(2.7)	20.8(4.4)	-−1.0/-0.7	−1.5
WHOQOL Psychological health	15.7(3.7)	19.2(5.7)	−1.0/-0.6	−2.1*
WHOQOL Social relationships	5.3(1.5)	8.7(1.7)	−2.3/-0.7	−2.2*
WHOQOL Environment	19.6(3.4)	24.7(2.4)	−1.7/-1.0	−2.0*
ASQ subscale Concealing	25.9(6.3)	23.7(6.2)	0.3/0.4	−1.4
ASQ subscale Adjusting	20.3(4.0)	17.6(6.0)	0.5/0.6	−1.9
ASQ subscale Tolerating	8.4(1.6)	13.0(2.4)	−2.2/-1.3	−2.4**
ERS	6.5(4.9)	13.5(5.3)	−1.7/-0.9	−2.0*

Notes: GHQ-28 = General Health Questionnaire 28; PCL-5 = Posttraumatic Checklist for DSM-5; PHQ-9 = Patient Health Questionnaire 9; SSS-8 = Somatic Symptom Scale 8; WHOQOL = World Health Organization Quality of Life; ASQ = Affective Style Questionnaire; ERS = Emotion Regulation Scale.

* *p* < .05, ** *p* < .025.

In the completer analysis, large effect sizes were seen for the GHQ-28 (*d* = 2.0) and WHOQOL scales (*d* = 1.0–2.3) (see Table 2). The changes in the PHQ-9 and the PCL-5 were not statistically significant on the Wilcoxon test, but showed moderate to large effect sizes (*d* = 0.6 and *d* = 0.8, respectively). The GHQ-28 subscales showed reductions with large effect sizes. Statistically significant reductions on the Wilcoxon test were observed in the GHQ-28 subscales anxiety/insomnia, social dysfunction, and severe depression. In addition, significant increases with large effect sizes were found in the WHOQOL subscales Psychological health, Social relationships, and Environment. No statistically significant changes were found in the WHOQOL subscale physical health, in the GHQ-28 subscale somatic symptoms, and in the somatic symptom scale (SSS-8), although large effect sizes were seen on the WHOQOL and GHQ scale.

For the GHQ as the main outcome criteria, we also computed the rate of clinical significant change (Jacobson et al., 1984). None of the participants reached the remission criterion of GHQ<6 (binary scored; Kalafi et al., 2002); however, five participants met the reliable change criterion, dropping from pre- to post-assessment of >7.4 (α = 0.05; reliability = 0.90, *SD*pre = 8.4). With respect to measures assessing possible mediators of improvement, in the completer analysis we found a large effect size for the affective style tolerating subscale (*d* = 2.2) and for the Emotion Regulation Scale (*d* = 1.7), both of which were significant by Ranked T test.

### 4.1. Feasibility

Feasibility of the programme was indicated by the high rate of session attendance. Out of the seven participants who completed treatment, three participated in 11, two in 10, and two in eight sessions. Drop-outs were not related to exposure components such as hyperventilation experiment; both drop-outs occurred prior to the exposure session. Significant barriers to participation and continuous attendance, however, were time overlap with regular German lessons and appointments as well as attorneys and counsellors due to official processing of asylum status, and so too increased distress and fear of being deported. Promoters of attendance were having native-speaking therapists and assessors facilitate communication, as compared to work with interpreter. Treatment was also facilitated by having therapists with knowledge about housing conditions and the asylum procedure; this created understanding of the participants, resulting in increased trust and openness towards the treatment.

At the end of treatment, participants were asked which components were helpful. Many participants (*n* = 5) said that it was helpful to speak their mother tongue. Most of the participants (*n* = 6) reported the body-focused stretching practices, the ‘bagh’ imagination, and the anxiety protocol to be very helpful, and the majority (*n* = 5) to be able to talk about daily problems like housing and the asylum procedure. Most of the participants also suggested the programme to be available for all people who have similar problems because they can encounter ‘fellow sufferers and support each other’. One participant said: ‘I was sceptical in the beginning, I didn’t believe in the treatment to be helpful. But then I saw how some other in the group begin to feel better and I tried to practice the things we talked about at home and it worked out. Yet, it is important that there is no pressure. It’s important to want it by yourself’. One participant expressed the helpful support of the group as follows: ‘The most important thing was not being alone’.

To sum up, the participants’ feedback indicated that the programme was feasible and acceptable with respect to language and cultural aspects, and that treatment was facilitated by doing in a group format that provided social support and other benefits.

## 5. Discussion

The present pilot study investigated the feasibility and effectiveness of a culturally adapted 12-week CBT group programme for Afghan refugees with mental health problems. CA CBT has been evaluated for various ethnic groups but not for Afghan refugees (Hinton et al., 2005, 2009; Jalal et al., 2017), and to our knowledge this is the first treatment study with a Farsi-speaking refugee sample outside Afghanistan. Due to specific cultural barriers in the utilization of psychotherapy (Alemi et al., 2016; Yaser et al., 2016) and the high comorbidity of PTSD with other trauma-related disorders (Alemi et al., 2014) in refugees, CA CBT is conceptualized as a low-threshold transdiagnostic treatment programme.

Our results indicate significant and strong reductions in depression and anxiety, accompanied by particularly great improvements in quality of life. As compared to trauma-focused treatments, such as Narrative Exposure Therapy (Neuner et al., 2010), and in line with the transdiagnostic concept of the treatment, improvement in the total score of the GHQ-28 was much larger than in the PLC-5, suggesting that the primary effect of the treatment may have been stronger on general mental health than on specific symptoms of PTSD. The reduction of the PCL-5 was lower than the controlled effects of RCTs with trauma-focused treatments in a recent meta-analysis, among them Narrative Exposure Therapy as the best-evaluated (Nosè et al., 2017). However, the large effect size and the statistical trend in our study in the PCL-5 suggest that, given an adequate sample size, the reduction in PTSD symptoms would have reached statistical significance.

There were large reductions in the GHQ-28 subscales anxiety/insomnia and severe depression. On the other hand, we didn’t observe changes that reached statistical significance on the Wilcoxon test in those measures that assess somatic symptoms (GHQ-28 subscale somatic symptoms, WHOQOL physical pain, Somatic Symptoms Scale), though large effect sizes were seen on the GHQ and WHOQOL scales. The GHQ has been found to be significantly correlated with depression and anxiety, thus representing somatic symptoms of mental disorders (Werneke, Goldberg, Yalcin, & Ustun, 2000), whereas the SSS-8 refers to cardiopulmonary, gastrointestinal, and pain symptoms that might be relevant for the assessment of general physical health, and possibly somatoform disorders (Gierk et al., 2014). Although the group programme also targets somatic symptoms, the failure to provide greater relief from somatic symptoms may be explained in part by persisting injuries as a long-term consequence of torture in three participants. But given the large effect sizes on two measures of somatic distress, with a large sample, statistical significance would in all likelihood have been observed.

Some findings of the current study suggest that CA CBT may increase the ability to adopt an accepting attitude towards negative thoughts and emotions, as reflected by the large effect observed in the ASQ-subscale tolerating (*d* = 2.2). In addition, the ERS scale greatly improved (*d* = 1.7), and of note the ERS is mainly a measure of ability to distance from negative affect. Although neither the sample size nor the timing of measurements allow for valid conclusions about mediation effects, it may be speculated whether acceptance may represent a potential mediator of the effects of our treatment. This would be in line with recent results from trials with patients suffering from anxiety disorders, showing that acceptance is a significant mediator of change in CBT (e.g. Arch et al., 2012). Relatedly, the ERS finding suggests a great improvement in ability to emotionally regulate as a key mediator of improvement (Hinton et al., 2009).

Another interesting finding is that participants reported considerable improvement of social functioning, as reflected in a significant decrease of GHQ-28 subscale social dysfunction (*d* = 1.0). This effect could be explained by providing social support and encouraging participants to have exchange on the stressors experienced in their current life with others within and outside the group. In addition, by providing psychoeducation about the nature of the consequences of traumata, this might also have provided relief from dysfunctional interpretations of emotional symptoms of trauma-related mental health problems. Thus, participants may have experienced a reduction in dysfunctional meta-beliefs about their problems (Yaser et al., 2016) that contribute to problems in interpersonal relationships, such as social avoidance and retreat.

Given the unstable life situation of refugees in the current study with respect to accommodation and asylum status, the drop-out rate (two out of nine) was lower than expected. This may be due to the growing group coherence among the participants. Another factor that may have increased motivation for treatment could be the fact that the therapists shared language and cultural background, thus bridging the gap when referring to difficult emotional issues. In addition, the therapists may have served as successful models for migrants who achieved acculturation and integration in German society. Of note, however, two patients dropped out. It is possible that dropouts may have been triggered by treatment sessions, but both patients entered treatment after session 1 (session 2 and session 5), thus not adhering to the treatment as delivered. Furthermore, in both sessions patients were not exposed to interventions that may trigger PTSD or panic.

There are several limitations to the present pilot study that should be mentioned. First, the lack of a control group impedes clear conclusions on alternative factors that may account for the reduction of psychopathological symptoms such as changes in the life situation, natural changes of the mental health problems, and unspecific influences such as attention and positive expectations about the treatment. Second, due to the small sample size, external validity is severely threatened: participants were male and relatively educated, so studies will need investigating efficacy in Afghan woman and the less educated. Third, adaptation to the specific cultural background of the participants was not based on systematic qualitative analyses of interviews, but rather on the status of current research, personal opinions of the therapists with migration background, and data from the interviews with individual participants. Fourth, we did not assess treatment integrity and, although weekly supervision was given by the senior author, and the second author had several years of experience in training of trauma counselling, the therapists had no previous experience with the treatment manual. Fifth, it is to be questioned if the subjects who agreed to participate are representative for the population. One could argue that only participants being open to psychotherapy tend to participate in group treatment, so that Afghan or Iranian refugees being sceptical towards psychotherapy may have been overlooked. This should be assessed in further studies. To sum up, despite the methodological shortcomings, the results of this pilot study are encouraging and indicate substantial changes in mental health symptoms, quality of life, and general emotion regulation processes. CA CBT appears to be a promising transdiagnostic treatment that may be appropriate as an initial low-threshold, easy accessible and feasible therapy in a stepped care approach. Results were particularly impressive since treatment was conducted in a group setting, which also increases scalability. However, further studies using randomized controlled trials with larger sample sizes are needed to test the efficacy of CA CBT in treating mental health problems of Afghan refugees.
